# Prevention of methamphetamine-induced microglial cell death by TNF-α and IL-6 through activation of the JAK-STAT pathway

**DOI:** 10.1186/1742-2094-9-103

**Published:** 2012-07-06

**Authors:** Vanessa Coelho-Santos, Joana Gonçalves, Carlos Fontes-Ribeiro, Ana Paula Silva

**Affiliations:** 1 Laboratory of Pharmacology and Experimental Therapeutics, Faculty of Medicine, University of Coimbra, Coimbra, Portugal; 2 Institute of Biomedical Research on Light and Image (IBILI), Faculty of Medicine, University of Coimbra, Coimbra, Portugal

**Keywords:** Apoptosis, Interleukine-6, JAK-STAT3, Methamphetamine, Microglia, Tumor necrosis factor-alpha

## Abstract

****Background**:**

It is well known that methamphetamine (METH) is neurotoxic and recent studies have suggested the involvement of neuroinflammatory processes in brain dysfunction induced by misuse of this drug. Indeed, glial cells seem to be activated in response to METH, but its effects on microglial cells are not fully understood. Moreover, it has been shown that cytokines, which are normally released by activated microglia, may have a dual role in response to brain injury. This led us to study the toxic effect of METH on microglial cells by looking to cell death and alterations of tumor necrosis factor-alpha (TNF-α) and interleukine-6 (IL-6) systems, as well as the role played by these cytokines.

****Methods**:**

We used the N9 microglial cell line, and cell death and proliferation were evaluated by terminal deoxynucleotidyl transferase dUTP nick end labeling assay and incorporation of bromodeoxyuridine, respectively. The TNF-α and IL-6 content was quantified by enzyme-linked immunosorbent assay, and changes in TNF receptor 1, IL-6 receptor-alpha, Bax and Bcl-2 protein levels by western blotting. Immunocytochemistry analysis was also performed to evaluate alterations in microglial morphology and in the protein expression of phospho-signal transducer and activator of transcription 3 (pSTAT3).

****Results**:**

METH induced microglial cell death in a concentration-dependent manner (EC_50_ = 1 mM), and also led to significant morphological changes and decreased cell proliferation. Additionally, this drug increased TNF-α extracellular and intracellular levels, as well as its receptor protein levels at 1 h, whereas IL-6 and its receptor levels were increased at 24 h post-exposure. However, the endogenous proinflammatory cytokines did not contribute to METH-induced microglial cell death. On the other hand, exogenous low concentrations of TNF-α or IL-6 had a protective effect. Interestingly, we also verified that the anti-apoptotic role of TNF-α was mediated by activation of IL-6 signaling, specifically the janus kinase (JAK)-STAT3 pathway, which in turn induced down-regulation of the Bax/Bcl-2 ratio.

****Conclusions**:**

These findings show that TNF-α and IL-6 have a protective role against METH-induced microglial cell death via the IL-6 receptor, specifically through activation of the JAK-STAT3 pathway, with consequent changes in pro- and anti-apoptotic proteins.

## **Background**

Methamphetamine (METH) is a potent addictive psychostimulant drug that easily crosses the blood–brain barrier and induces severe brain damage, leading to neurological abnormalities and eventually to psychiatric disorders. Several studies have demonstrated that people who misuse METH reveal deficits in the dopaminergic and serotonergic systems, hippocampal volume reduction, white-matter hypertrophy and microglia activation [1-3]. However, the underlying mechanisms of its toxicity remain to be fully determined. Nevertheless, oxidative stress [4,5], excitotoxicity [6,7], mitochondrial dysfunction [5,8], and microgliosis [9,10] are some features of METH neurotoxicity. Recently, our group have demonstrated that a single high dose of METH (30 mg/kg by intraperitoneal injection (i.p.)) triggered a neuroinflammatory response in mouse hippocampus, characterized by the activation of microglia and production of proinflammatory cytokines, namely TNF-α [9,11] and IL-6 [11]. In agreement, Thomas *et al.*[12] showed that microglial activation is a specific marker for METH neurotoxicity being linked to dopamine or serotonin (5-hydroxytryptamine) nerve terminal damage. However, the attenuation of microglial activation is not by itself sufficient to protect against METH-induced striatal dopaminergic neurotoxicity [13], and this lack of neuroprotection was shown to be due to the inability of minocycline to modulate TNF-α signaling. Moreover, Thomas and collaborators [14] concluded that microglial-specific fractalkine receptor (CX3CR1) signaling does not modulate METH neurotoxicity or microglial activation.

It is known that after a central nervous system injury, microglial cells became activated and, besides morphological alteration, they get the capacity to produce and release high levels of proinflammatory cytokines [9,15]. These high levels could cause the microglial cells to shift from having a beneficial role to a detrimental one [16]. Moreover, the action of some cytokines can stimulate the synthesis and function of others, resulting in a complex pathway called cytokine cascade [17]. Specifically, TNF-α has been reported as a potent stimulator of IL-6 production [18,19], whose pleiotropic action can be through TNF receptor 1 (TNFR1/p55) or 2 (TNFR2/p75) [20]. The activation of TNF receptors stimulates several signaling pathways that regulate cellular processes, ranging from cell proliferation and differentiation to cell death [21]. Regarding IL-6, its production seems to be regulated by several signaling cascades [18,22], including by TNF-α mainly via the nuclear factor kappa-light-chain-enhancer of activated B cells (NF-κB) pathway [23,24]. The growing interest in central IL-6 is in part owing to its involvement in the neuroinflammatory response [25] and neurotropic processes [26-28], as well as in several brain pathologies [29,30]. This pleiotropic cytokine acts through two receptors, the IL-6 receptor-alpha (IL-6R-α, also known as gp80 or CD126) and a soluble form of the IL-6R [31]. When IL-6 binds to its receptor, homodimerization of gp130 occurs, followed by the activation of associated janus kinases (JAKs) [32], and the recruitment of signal transducer and activator of transcription (STAT) proteins to the nucleus, where they will modulate gene transcription [33]. *In vitro* and *in vivo* studies showed that IL-6 signaling in the central nervous system is carried out by STAT3 that is phosphorylated by JAK at Tyr705 [34,35].

Regarding the effect of METH on proinflammatory cytokines, Ladenheim *et al.*[36] showed that the IL-6 null genotype affords protection to dopamine and serotonin terminal damage, apoptotic cell death, and reactive gliosis induced by METH (four i.p. injections of 5 or 10 mg/kg). More recently Tocharus *et al.*[37] reported that METH reduced rat microglial cells viability simultaneously with the increase of IL-6 and TNF-α expression and the production of both reactive oxygen species and reactive nitrogen species, suggesting that cytokines may also participate in METH toxicity. Despite these pieces of evidence, it remains to be clarified whether neuroinflammation and the consequent synthesis and release of proinflammatory cytokines is a cause or consequence of the neurotoxicity induced by METH.

The present study aimed to determine whether METH exerts a direct effect on microglial cells and to unravel the beneficial or detrimental role of IL-6 and TNF-α. We found that METH induces microglial cell death, and also affects microglial morphology and proliferation. Additionally, this drug increased the protein levels of both cytokines and respective receptors. Moreover, the release of TNF-α and IL-6 observed after METH insult was shown to be a consequence of METH toxicity and not a cause. Interestingly, we also demonstrated that exogenous low levels of both cytokines have a protective role against METH toxicity through activation of the IL-6/JAK-STAT3 signaling pathway and, consequently, alterations in the levels of pro- and anti-apoptotic proteins. The present work allows us to better understand how METH affects the microglia dynamics and suggest that the IL-6 system is an important target to prevent, or at least to minimize, the toxic effects of METH.

## **Methods**

### **Cell culture**

The murine microglial cell line N9 (kindly provided by Prof. Claudia Verderio, CNR Institute of Neuroscience, Cellular and Molecular Pharmacology, Milan, Italy) was obtained by immortalization of E13 mouse embryonic brain cultures with the 3RV retrovirus carrying an activated v-myc oncogene [38]. Cells were cultured in Roswell Park Memorial Institute medium (RPMI; Gibco, Paisley, UK) supplemented with 5% Fetal Bovine Serum (FBS; Gibco), 23.8 mM sodium bicarbonate (Sigma-Aldrich, St. Louis, MO, USA), 30 mM D-Glucose (Sigma-Aldrich), 100 U/mL penicillin and 100 μg/mL streptomycin (Gibco), and were maintained at 37 °C, 95% air and 5% CO_2_ in a humidified incubator. N9 cells were then seeded onto 24-well plates with 1.6 × 10^4^ cells/well for terminal deoxynucleotidyl transferase dUTP nick end labeling (TUNEL) assay, 5-bromo-2’-deoxyuridine (BrdU) incorporation and immunocytochemistry; 12-well plates with 5.6 × 10^4^ cells/well for ELISA; and 6-well plates with 5 × 10^5^ cells/well for western blotting.

### **TUNEL assay**

N9 cells were incubated with increasing concentrations (0.1 to 4 mM) of METH ((+)-methamphetamine hydrochloride; Sigma-Aldrich) for 24 h. The present concentration range of METH was chosen based on previous *in vitro* studies [37,39,40]. In order to confirm cell death by apoptosis, microglial cells were co-incubated for 24 h with 1 mM METH plus z-Val-Ala-DL-Asp (OMe)-fluoromethylketone (Z-VAD; Calbiochem, Nottingham, UK) at a concentration of 25 μM that was chosen based on prior works developed by our group [41,42].

To investigate the contribution of endogenous and exogenous TNF-α, N9 cells were co-incubated with 100 μg/mL anti-TNF-α antibody (Upstate Biotechnology, Inc., Lake Placid, NY, USA) or 1 ng/mL TNF-α (R&D systems, Abingdon, UK) plus 1 mM METH over 24 h. The role played by endogenous IL-6 in METH-induced cell death was investigated by pre-exposing the cells to 10 μg/mL anti-IL-6R antibody (R&D systems) for 20 min or 20 μM AG490 (Calbiochem) for 1 h, and then co-incubated with 1 mM METH. To analyze the effect of exogenous IL-6, cells were co-exposed to 1 ng/mL IL-6 (R&D systems) plus 1 mM METH for 24 h, in the absence or presence of IL-6R antibody or 20 μM AG490, as mentioned above. Moreover, in an attempt to clarify the crosstalk between these cytokines in METH-induced apoptosis, N9 cells were pre-incubated for 20 min with IL-6R antibody and then co-incubated for 24 h with 1 ng/mL TNF-α plus 1 mM METH. Anti-TNF-α antibody and anti-IL-6R antibody were used at 100 μg/mL or 10 μg/mL to neutralize the effects of 1 ng/mL TNF-α [41,43] or 1 ng/mL IL-6, respectively (in agreement with the instruction of the supplier). Tyrphostin AG 490 has been successfully used to inhibit the activation of STAT3 in microglia cells [44,45], and we chose the concentration of 20 μM based on previous studies [45,46].

After the respective treatments, we collected the supernatant that contained cells that had detached from the bottom of the wells (dead or dying cells). Adherent cells (surviving cells) were trypsinized and added to the detached cells in order to obtain the whole population of cells. Then, microglial cells were fixed with 4% paraformaldehyde (PFA) and adhered to superfrost microscope slides (Thermo Scientific, Menzel GmbH & Co KG, Braunschweig, Germany) by centrifugation (113 × *g*, 5 min; Cellspin I, Tharmac GmbH, Waldsolms, Germany). Apoptotic cell death was further evaluated by the TUNEL assay (Roche Diagnostics GmbH, Mannheim, Germany), as follows. Cells were rinsed with 0.01 M PBS (137 mM sodium chloride, 2.7 mM potassium chloride, 4.3 mM disodium hydrogen phosphate, 1.47 mM monopotassium dihydrogen phosphate, pH 7.4), permeabilized in 0.25% Triton X-100 for 30 min at room temperature (RT), and incubated with terminal deoxynucleotidyl transferase buffer for 1 h at 37 °C in a humidified chamber. Afterwards, N9 cells were washed in terminal buffer (300 mM sodium chloride and 30 mM sodium citrate) for 15 min and in 0.01 M PBS for 5 min. Incubation with fluorescein Avidin D (1:100; Vector Laboratories, Burlingame, CA, USA) was performed for 1 h, followed by nuclei counterstaining with 5 μg/ml Hoechst 33342 (Sigma-Aldrich) for 5 min. The slides were mounted in Dako fluorescent medium (Dako North America Inc., Carpinteria, CA, USA) and fluorescent images for cell counts were recorded using an Axiovert 200 M fluorescence microscope (Carl Zeiss, Oberkochen, Germany).

### **Immunocytochemistry**

Microglial cells were exposed to 1 mM METH for 24 h and then rinsed with 0.01 M PBS, fixed with 4% PFA for 30 min at RT, permeabilized with acetone for 3 min at −20 °C and blocked with 0.01 M PBS containing 10% FBS for 1 h at RT. Afterwards, cells were incubated with the polyclonal antibody ionized calcium binding adaptor molecule-1 (Iba-1; 1:400; Abcam, Cambridge, MA, USA) overnight at 4 °C and then incubated with Alexa Fluor 488 anti-goat (1:200; Invitrogen, Paisley, UK) together with rhodamine phalloidin (1:200, Molecular Probes, Invitrogen) for 1 h 30 min at RT, which allowed the visualization of F-actin filaments. To evaluate STAT3 activation, cells were exposed to 1 mM METH alone or co-exposed with 1 ng/mL IL-6 for 24 h. After treatment, cells were rinsed with 0.01 M PBS, fixed with 4% PFA for 30 min at RT, permeabilized with 0.5% Triton X-100 for 30 min at RT, blocked with PBS containing 1% BSA for 1 h at RT, and incubated with the monoclonal antibody phosphorylated (p)-STAT3 (1:100; Cell Signaling Technology, Inc., Danvers, MA, USA) overnight at 4 °C. Cells were then incubated with Alexa Fluor 594 donkey anti-mouse (1:200; Invitrogen) for 1 h 30 min at RT and stained with Hoechst 33342 (4 μg/mL; Sigma-Aldrich) for 5 min at RT in the dark. Finally, cultures were mounted in Dako fluorescence medium (Dako North America Inc.) and images were captured using a LSM 710 Meta confocal microscope (Carl Zeiss, Göttingen, Germany).

### **Cell proliferation studies**

Cell proliferation was evaluated by BrdU (Sigma-Aldrich) incorporation based on previous work [47]. Microglial cells were treated with METH (0.001 to 1 mM) and/or 25 μM Z-VAD for 24 h; 10 μM BrdU was added in the last 2 h of the culture session. Cells were then fixed in 4% PFA for 30 min and rinsed in 0.01 M PBS. BrdU was unmasked by successive passages in 1% Triton X-100 for 30 min, ice-cold 0.1 M hydrogen chloride for 20 min, and 2 M hydrogen chloride for 40 min at 37 °C, following neutralization with 0.1 M hydrous sodium borate buffer (pH 8.5; Sigma-Aldrich) for 10 min at RT and incubation in a blocking solution with 3% BSA (Sigma-Aldrich) and 0.3% Triton X-100 in 0.01 M PBS for 30 min at RT. Afterwards, microglial cells were incubated with rat anti-BrdU (1:100; AbD Serotec, Oxford, UK) in 0.01 M PBS containing 0.3% Triton X-100 and 0.3% BSA, overnight at 4 °C, and then with Alexa Fluor 488 (1:200; Invitrogen) for 1 h 30 min at RT, followed by cell nuclei counterstaining with 4 μg/ml Hoechst 33342 for 5 min at RT. Cells were mounted in Dako fluorescent medium (Dako North America Inc.) and images were recorded using a camera Leica DMIRE2 incorporated on a fluorescence microscope (Leica CTRMIC; Leica Microsystems, Wetzlar, Germany).

### **Enzyme-linked immunosorbent assay**

To evaluate the intracellular and extracellular contents of TNF-α and IL-6, cells were treated with 1 mM METH or 1 μg/mL lipopolysaccharide (LPS; positive control) for 1 h or 24 h followed by ELISA assay (Bender MedSystem®, Vienna, Austria). For that purpose, the supernatant was removed and centrifuged for 15 min at 17,968 × *g* at 4 °C, and then cells were lysed using a specific buffer (pH 8.0) as follows: 150 mM sodium chloride, 10 mM Tris-hydrogen chloride, 10% Triton X-100, 1 mM ethylenediaminetetraacetic acid complemented by a protease inhibitor cocktail tablet (Roche Applied Sciences, Basel, Switzerland). Cells were then sonicated, and protein concentration was determined by the bicinchoninic acid method, and stored at −20 °C until further use. ELISA assay was then performed according to manufacturers' instructions. Briefly, 96-well microtiter plates were coated with capture antibody (5 μg/mL), sealed and left overnight at 4 °C. Then, wells were washed with 0.01 M PBS plus 0.05% Tween 20, blocked with 0.01 M PBS plus 0.5% BSA and 0.05% Tween 20, and left overnight at 4 °C. Next, N9 cell culture samples and biotin-conjugated antibodies (1:1,000) were added to all wells, and incubated at RT for 2 h on a microplate shaker (200 rpm). After washing, streptavidin-horseradish protein (1:5,000) was added and kept once again at RT on a microplate shaker (200 rpm) for 1 h. After washing, tetramethylbenzidine substrate solution (eBioscience, Vienna, Austria) was added to each well for 10 to 20 min at RT. The reaction was stopped by adding 1 M phosphoric acid, and the absorbance was measured with a microplate reader (Biotek, Synergy HT, Winooski, USA), using a sample wavelength fixed at 450 nm and a reference wavelength at 655 nm. A standard curve for both cytokines was used to calculate the respective extracellular (pg/mL) and intracellular (pg/mg of total protein) protein levels.

### **Western blot analysis**

Cells were exposed for 1 h or 24 h to 1 mM METH, co-exposed with 1 mM METH and 1 ng/mL IL-6 for 24 h, or pre-exposed with 20 μM AG 490 for 1 h followed by incubation with 1 mM METH for 24 h. After treatment, cells were lysed on ice in radioimmunoprecipitation assay (RIPA) buffer (0.15 M sodium chloride, 0.05 M Tris-base, 0.005 M ethyleneglycoltetraacetic acid, 0.5% sodium deoxycholate, 0.1% SDS and 1% X-Triton, pH 7.5) supplemented with protease inhibitor cocktail tablets (Roche Applied Sciences), quantified using the bicinchoninic acid method, and stored at −20 °C until further use. Total proteins (TNFR1, 40 μg; IL-6R-α, 25 μg; Bcl-2 and Bax, 30 μg) were separated by electrophoresis on SDS polyacrylamide gel, transferred onto polyvinylidene difluoride membrane (Millipore, Madrid, Spain), and then blocked with 5% non-fat milk (TNFR1, IL-6R-α and Bcl-2 proteins) or 4% BSA (Bax protein) for 1 h at RT. Afterwards, the membranes were incubated overnight at 4 °C with the primary antibodies as follows: rabbit anti-TNFR1 (1:200, Santa Cruz Biotechnology, Santa Cruz, CA,USA), goat anti-IL-6R-α (1:500, R&D Systems), mouse anti-Bcl-2 (1:200, Santa Cruz Biotechnology) and rabbit anti-Bax (1:100, Santa Cruz Biotechnology). Then, membranes were washed and incubated for 1 h at RT with alkaline phosphatase conjugated secondary antibody (anti-goat and anti-mouse-1:10,000; anti-rabbit-1:20,000; Amersham, GE Healthcare Life Science, Little Chalfont, Buckinghamshire, UK) and visualized using ECF reagent (Amersham) on Typhoon FLA 9000 (GE Healthcare Bio-Sciences AB, Uppsala, Sweden). Immunoblots were reprobed with β-actin antibody (1:10,000, Sigma-Aldrich) to ensure equal sample loading, and densitometric analyses were performed using the ImageQuant version 5.0 software.

### **Statistical analysis**

Results are expressed as mean ± standard error of the mean (SEM). Data were analyzed using the one-tailed Mann–Whitney test for comparison between two groups, or multiple level analysis of variance (ANOVA) followed by Dunnett’s or Bonferroni’s *post hoc* test, as indicated in figure legends. All statistics were calculated using GraphPad Prism 5.0 (GraphPad Software, San Diego, CA, USA). The level of significance was *P* <0.05. For the quantification of TUNEL and BrdU-positive cells, six independent microscopy fields per coverslip with 200× or 400× magnification were acquired, respectively, and results are expressed as percentages of total cells stained with Hoechst 33342 per each field (n = number of fields). For western blot and ELISA assay, n corresponds to the number of independent experiments.

## **Results**

### **Effect of METH on microglia: Cell death, morphological changes and proliferation**

**Figure 1:**
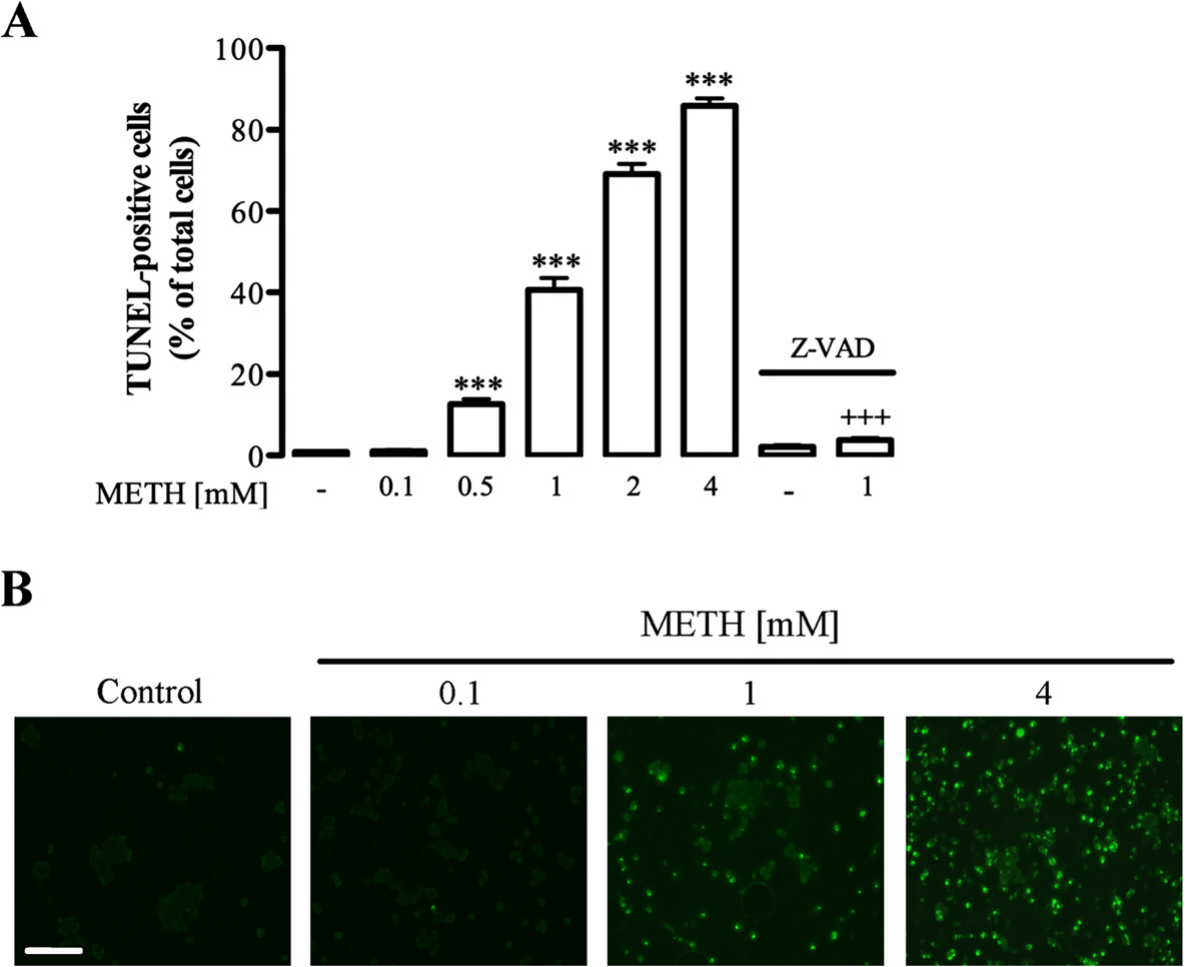
**METH induces microglial cell death. (A)** METH increased the number of TUNEL-positive cells in a concentration-dependent manner (0.1 to 4 mM for 24 h), and Z-VAD (25 μM) completely prevented the apoptotic cell death induced by 1 mM METH. The results are expressed as percentage of total cells ± SEM (n = 10 to 25). ^***^P <0.001, Dunnett’s multiple comparison test, significantly different when compared to control. ^+++^*P* <0.001 to Bonferroni’s multiple comparison test, significantly different comparing with 1 mM METH. **(B)** Representative fluorescence images of TUNEL-positive cells following treatment with 0.1, 1 or 4 mM METH. Scale bar, 20 μm. METH: methamphetamine; SEM: standard error of the mean; TUNEL: terminal deoxynucleotidyl transferase mediated dUTP nick end labeling assay; Z-VAD: z-Val-Ala-DL-Asp (OMe)-fluoromethylketone.

It has been previously suggested that microglia activation contributes to METH-related neuropathology [10], but there is no evidence concerning the direct effect of this drug on microglial cells. Thus, in the present study we aimed to clarify if METH induces microglial cell death. For that purpose, we exposed N9 microglial cells to different concentrations of METH (0.1 to 4 mM) over 24 h, and apoptotic cell death was evaluated by TUNEL assay. We observed that METH significantly increases the number of apoptotic cells in a concentration-dependent manner(Figure 1A, B). Specifically, at a lower concentration there was no significant difference when compared to control (control: 0.90 ± 0.12%, n = 49; 0.1 mM METH: 1.06 ± 0.28% of total cells, n = 10; Figure 1A). However, at concentrations above 0.5 mM there was an increase of cell death as follows: 0.5 mM: 12.63 ± 1.21% of total cells, n = 10; 1 mM: 36.44 ± 1.21%, n = 37; 2 mM: 69.12 ± 2.53%, n = 10; 4 mM: 85.75 ± 1.84% of total cells, n = 10 (Figure 1A, B). Based on these results, we chose to use 1 mM of METH (EC_50_) in the subsequent studies reported in this manuscript. Moreover, in order to confirm that METH triggers microglial apoptosis, cells were co-exposed to the drug (1 mM) plus Z-VAD (25 μM), a cell-permeant pan caspase inhibitor. We concluded that Z-VAD alone did not have any effect (2.14 ± 0.42% of total cells, n = 20; Figure 1A), but was able to completely prevent cell death induced by 1 mM METH (3.8 ± 0.53% of total cells, n = 20; Figure 1A).

**Figure 2:**
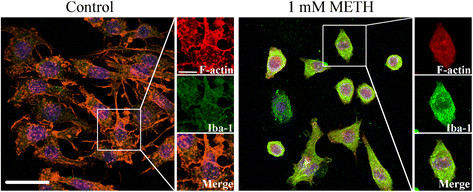
**METH causes microglial activation and cytoskeleton re-organization.** Representative confocal images of F-actin (red) and Iba-1 immunoreactivity (green) in N9 microglial cells under control conditions and exposed to 1 mM METH. Cells were also stained with Hoechst 33342 (blue). Scale bar, 20 μm and 50 μm. Iba-1: ionized calcium binding adaptor molecule-1; METH: methamphetamine.

To evaluate possible morphological changes in surviving cells, we performed Iba-1 and F-actin staining (Figure 2). Untreated N9 microglia cultures showed considerable ramifications, particularly lamellipodium- and filopodium-like structures, indicating a surveillance state. However, METH led to an arrangement of the actin cytoskeleton and cells acquired a round shape with retracted filopodia characteristic of microglia activation (Figure 2). In addition to these morphological alterations, we also observed an increase of Iba-1 immunoreactivity induced by METH (Figure 2).

**Figure 3:**
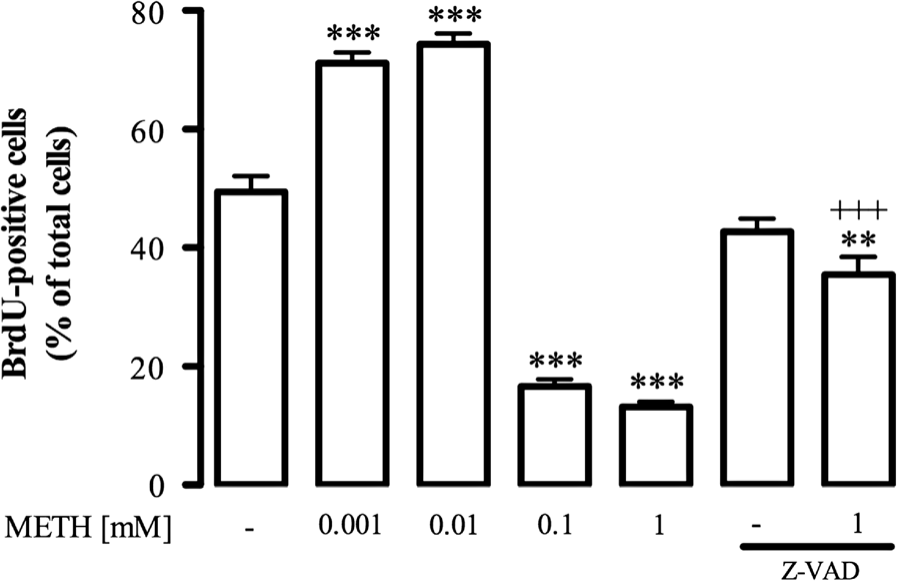
**METH affects microglial proliferation.** METH increased the number of BrdU-positive cells at 0.001 and 0.01 mM, but had a negative effect at 0.1 and 1 mM. Z-VAD (25 μM) reduced, but did not completely prevent, the decrease in the number of BrdU-positive cells induced by METH. The results are expressed as percentage of total cells ± SEM (n = 10 to 35). ^***^P <0.01, ^***^P <0.001, Dunnett’s multiple comparison test, significantly different comparing to control. ^+++^*P* <0.001, Bonferroni’s multiple comparison test, significantly different comparing with 1 mM METH. BrdU: 5-bromo-2’-deoxyuridine; METH: methamphetamine; SEM: standard error of the mean; Z-VAD: z-Val-Ala-DL-Asp (OMe)-fluoromethylketone.

The effect of METH on microglial proliferation was also studied. We found that low concentrations of METH (0.001 and 0.01 mM) increased microglia proliferation (control: 49.45 ± 2.65%, n = 33; 0.001 mM METH: 71.10 ± 1.80%, n = 23; 0.01 mM METH: 74.27 ± 1.83% of total cells, n = 24; Figure 3). On the other hand, higher concentrations of METH decreased cell proliferation, to 16.61 ± 1.20% (n = 21) with 0.1 mM METH and 13.15 ± 0.90% of total cells (n = 35) with 1 mM METH (Figure 3). It is noteworthy that 0.1 mM METH did not induce cell death, which demonstrates that METH also has a negative impact on microglial proliferation. Moreover, since 1 mM METH induced cell death we further co-exposed the cells with Z-VAD to clarify if the decrease in the number of BrdU-positive cells was due to cell death instead of a direct effect on proliferation. Interestingly, Z-VAD reduced, but not completely, the effect of METH (35.45 ± 3% of total cells, n = 10; Figure 3) showing that this toxic concentration negatively affects both cell viability and proliferation.

### **METH-induced alterations on microglial TNF-α and IL-6 systems**

**Figure 4:**
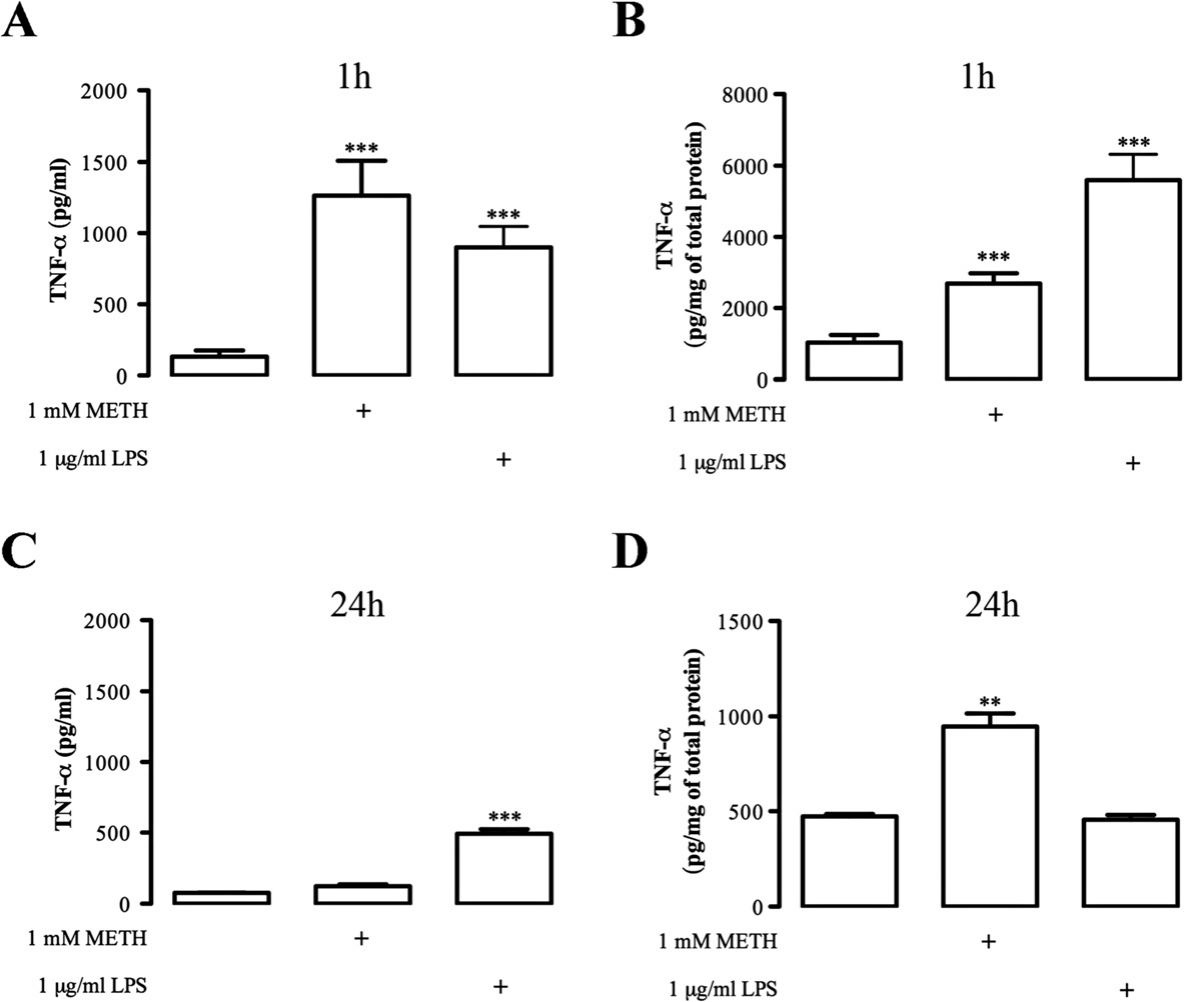
**METH triggers an early increase in TNF-α protein levels.** The effect of 1 mM METH on the **(A, C)** extracellular and **(B, D)** intracellular TNF-α levels was evaluated after **(A, B)** 1 h and **(C, D)** 24 h of drug exposure by ELISA. As a positive control we used 1 μg/mL LPS. Data are expressed as mean ± SEM of pg/mL for release and pg/mg of total protein for the intracellular levels (n = 3 to 13). ^**^P <0.01, ^***^P <0.001, Dunnett’s post-test, significantly different from control. ELISA: enzyme-linked immunosorbent assay; LPS: lipopolysaccharide; METH: methamphetamine; SEM: standard error of the mean; TNF-α: tumor necrosis factor-alpha.

Microglia activation by inflammatory stimuli increases the synthesis of proinflammatory cytokines such as TNF-α and IL-6 [48]. To evaluate the possible changes in microglial TNF-α and IL-6 systems triggered by METH, we measured the released and intracellular levels of these cytokines by ELISA at 1 h and 24 h after drug exposure. Regarding TNF-α, we observed that following 1 h of METH exposure, both extracellular (Figure 4A) and intracellular (Figure 4B) levels were significantly increased to 1,265 ± 244.4 pg/mL (n = 5) and 2,692.0 ± 287.3 pg/mg of total protein (n = 13) respectively when compared with control (extracellular: 132.2 ± 43.1 pg/mL, n = 8; intracellular: 1,032 ± 214.3 pg/mg of total protein, n = 7; Figure 4A, B). Moreover, after 24 h there were no changes in the extracellular levels (124.8 ± 12.36 pg/mL, n = 3; Figure 4C) when comparing to control (76.95 ± 1.76 pg/mL, n = 3; Figure 4C), but there was an increase of the intracellular levels (control: 473.60 ± 12.59 pg/mg total protein, n = 3; 1 mM METH: 946.4 ± 68.89 pg/mg total protein, n = 3; Figure 4D). As expected, 1 μg/mL LPS increased both extracellular (900.3 ± 146.4 pg/mL, n = 8; Figure 4A) and intracellular TNF-α levels (5,597 ± 726.4 pg/mg of total protein, n = 8; Figure 4B) at 1 h post-METH exposure, but after 24 h there was only a significant increase in the extracellular levels of TNF-α (492.3 ± 31.54 pg/mL, n = 3; Figure 4C).

**Figure 5:**
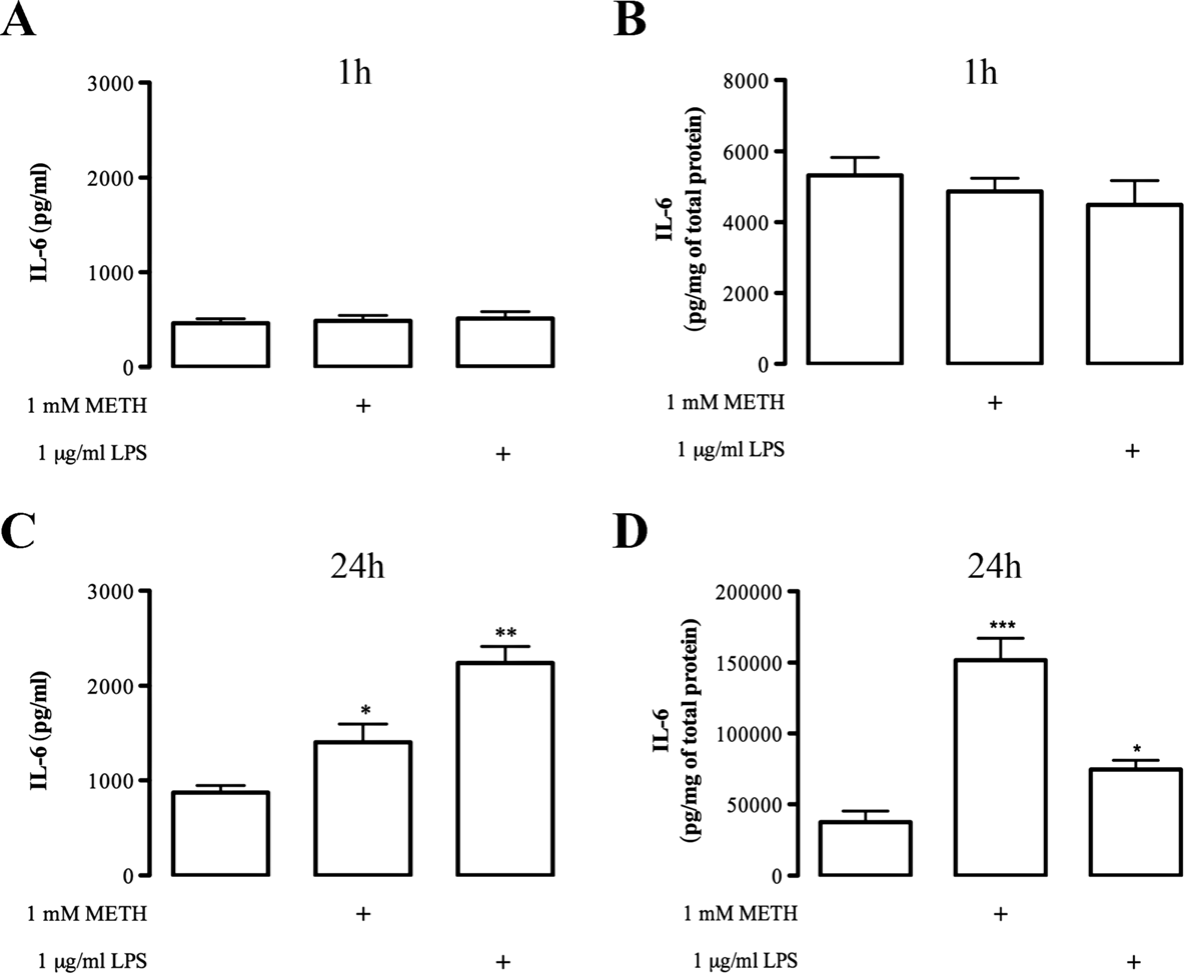
**METH increases IL-6 protein levels after 24 hours.** METH did not interfere with the **(A)** extracellular and **(B)** intracellular levels of IL-6 when analyzed 1 h post-treatment. However, following 24 h of METH exposure there was a significant increase in the **(C)** extracellular and **(D)** intracellular levels of IL-6. The treatment with (C, D) 1 μg/mL LPS was used as a positive control and up-regulated IL-6 levels after 24 h. Data are expressed as mean ± SEM of pg/mL for extracellular levels and pg/mg of total protein for intracellular levels (n = 4-15). ^*^P <0.05, ^**^P <0.01, ^***^P <0.001, Dunnett’s post-test, significantly different from control. IL-6: interleukin-6; LPS: lipopolysaccharide; METH: methamphetamine; SEM: standard error of the mean.

The analysis of IL-6 levels showed that neither the extracellular nor the intracellular content of this cytokine was changed by 1 mM METH at 1 h after exposure(Figure 5A, B). Extracellular levels were control: 460.8 ± 47.96 pg/mL, n = 15; METH; 486.6 ± 55.99 pg/mL, n = 13; and LPS; 507.6 ± 73.80 pg/mL, n = 15 (Figure 5A). The intracellular content was control: 5,321 ± 506.9 pg/mg, n = 8; METH: 4,867 ± 374.70 pg/mg, n = 9; and LPS: 4,487 ± 691.40 pg/mg, n = 8 (Figure 5B). On the contrary, after 24 h there was a significant increase of the release levels of IL-6 (Figure 5C) triggered by 1 mM METH (1,403 ± 192.80 pg/mL, n = 4), as well as by 1 μg/mL LPS (2,242 ±175.30 pg/mL, n = 4). Interestingly, there was also a very significant increase in the intracellular content of this cytokine at 24 h after METH exposure (151,711 ± 15,479 pg/mg of total protein, n = 4; Figure 5D). This also occurred, albeit at a lower scale, in the presence of 1 μg/mL LPS (74,502 ± 6,564 pg/mg of total protein, n = 6), when compared with the control (37,335 ± 7,877 pg/mg of total protein, n = 8; Figure 5D).

**Figure 6:**
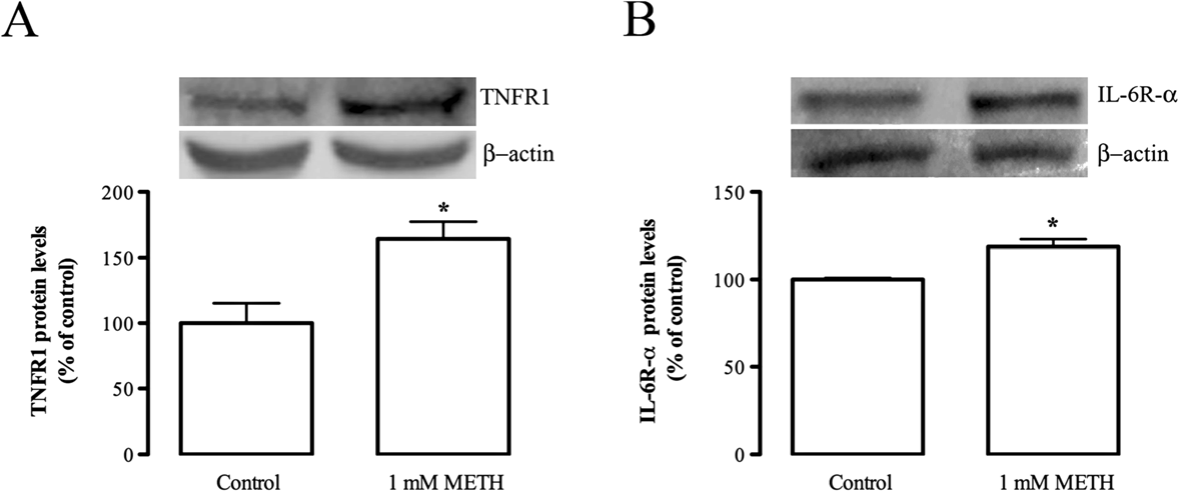
**METH increases TNFR1 and IL-6R-α protein levels.** Quantification of **(A)** TNFR1 and **(B)** IL-6R-α protein levels in N9 microglial cells at 1 h and 24 h post-METH exposure, respectively. Above the bars, representative western blot images of TNFR1 (55 kDa), IL-6R-α (63 kDa) and actin (42 kDa) are shown. The results are expressed as mean percentage of control ± SEM (n = 3 to 15). ^*^P <0.05, Mann–Whitney post-test, significantly different from control. IL-6R-α: interleukin-6 receptor-alpha; kDa: kiloDaltons; LPS: lipopolysaccharide; METH: methamphetamine; SEM: standard error of the mean; TNFR1: tumor necrosis factor receptor 1.

Since METH induced significant alterations in TNF-α and IL-6 protein levels, we further investigated if METH also interferes with the expression of the cytokine receptors. By western blot analysis, we could observe a significant increase in TNFR1 protein levels at 1 h post-METH (1 mM) exposure to 164.10 ± 13.04% of the control (n = 6; Figure 6A), but no differences were verified after 24 h (data not shown). Regarding IL-6R-α protein levels, we only found a significant increase in the protein levels of this cytokine at 24 h after drug treatment (118.6 ± 4.33% of the control, n = 15; Figure 6B), without alterations at 1 h (data not shown).

Here, we demonstrate that METH increases the extracellular and intracellular levels of TNF-α and IL-6 in N9 microglial cells, together with the up-regulation of the receptor protein levels, TNFR1 and IL-6R-α.

### **Effect of endogenous TNF-α and IL-6 on METH-induced microglial cell death**

**Figure 7:**
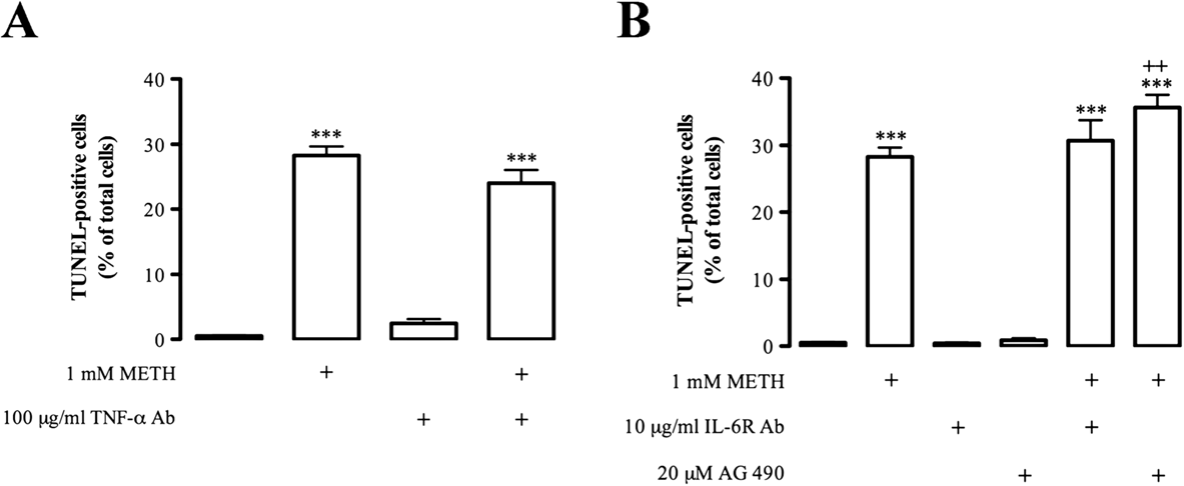
**Endogenous TNF-α and IL-6 are not involved in METH-induced microglial cell death.** The increase in the number of TUNEL-positive cells induced by 1 mM METH (24 h) was not changed by **(A)** TNF-α antibody or **(B)** IL-6R antibody, and the JAK-STAT pathway inhibitor (20 μ AG 490) exacerbated the toxic effect of METH. The results are expressed as mean percentage of total cells ± SEM (n = 17 to 84). ^***^P <0.001, Dunnett’s post-test, significantly different when compared with control. ^++^*P* <0.01, Bonferroni’s post-test, when compared with 1 mM METH. IL-6: interleukin 6; IL-6R: interleukin 6 receptor; JAK-STAT: janus kinase-signal transducer and activator of transcription; METH: methamphetamine; SEM: standard error of the mean; TNF-α: tumor necrosis factor-alpha; TNFR1: tumor necrosis factor receptor 1.

The role of cytokines such as TNF-α and IL-6 in response to brain injury remains unclear, in part due to its dual role [25,29,43]. Thus, since we had shown that METH increases TNF-α and IL-6 release, our next approach was to investigate the role of these endogenous cytokines under METH-induced cell death. For that, we quantified the number of TUNEL-positive cells in the presence of TNF-α (Figure 7A) or IL-6R (Figure 7B) neutralizing antibodies. We observed that 100 μg/mL TNF-α antibody was not able to prevent cell death induced by 1 mM METH (control: 0.5 ± 0.09%, n = 84; METH: 28.3 ± 1.41%, n = 86; METH + TNF-α antibody: 24.0 ± 2.05% of total cells, n = 23; Figure 7A). Moreover, TNF-α antibody *per se* did not induce microglial toxicity (2.5 ± 0.68% of total cells, n = 13; Figure 7A). Accordingly, the neutralization of endogenous IL-6 with IL-6R antibody (10 μg/mL) did not change the number of apoptotic cells induced by 1 mM METH alone (METH: 28.3 ± 1.41%; METH + IL-6R antibody: 31.7 ± 3.06% of total cells, n = 23; Figure 7B). Interestingly, the blockade of the JAK-STAT3 pathway by 20 μM AG 490, a tyrosine kinase inhibitor that blocks the autokinase activity of JAK and consequently the DNA binding of STATs [49], exacerbated the cell death induced by METH to 35.7 ± 1.91% of total cells (n = 18; Figure 7B). Moreover, IL-6R antibody or AG 490 *per se* did not induce significant cell death (IL-6R antibody: 0.4 ± 0.15%, n = 24; AG490: 0.86 ± 0.29% of total cells, n = 17; Figure 7B).

Overall, these results show that the blockade of endogenous TNF-α or IL-6 signaling pathways was not able to prevent cell death induced by METH, which suggests that the increase of TNF-α and IL-6 intracellular levels and their release are a consequence and not a cause of METH-induced microglia toxicity.

### **Protective effect of exogenous TNF-α and IL-6 against microglial cell death induced by METH**

**Figure 8:**
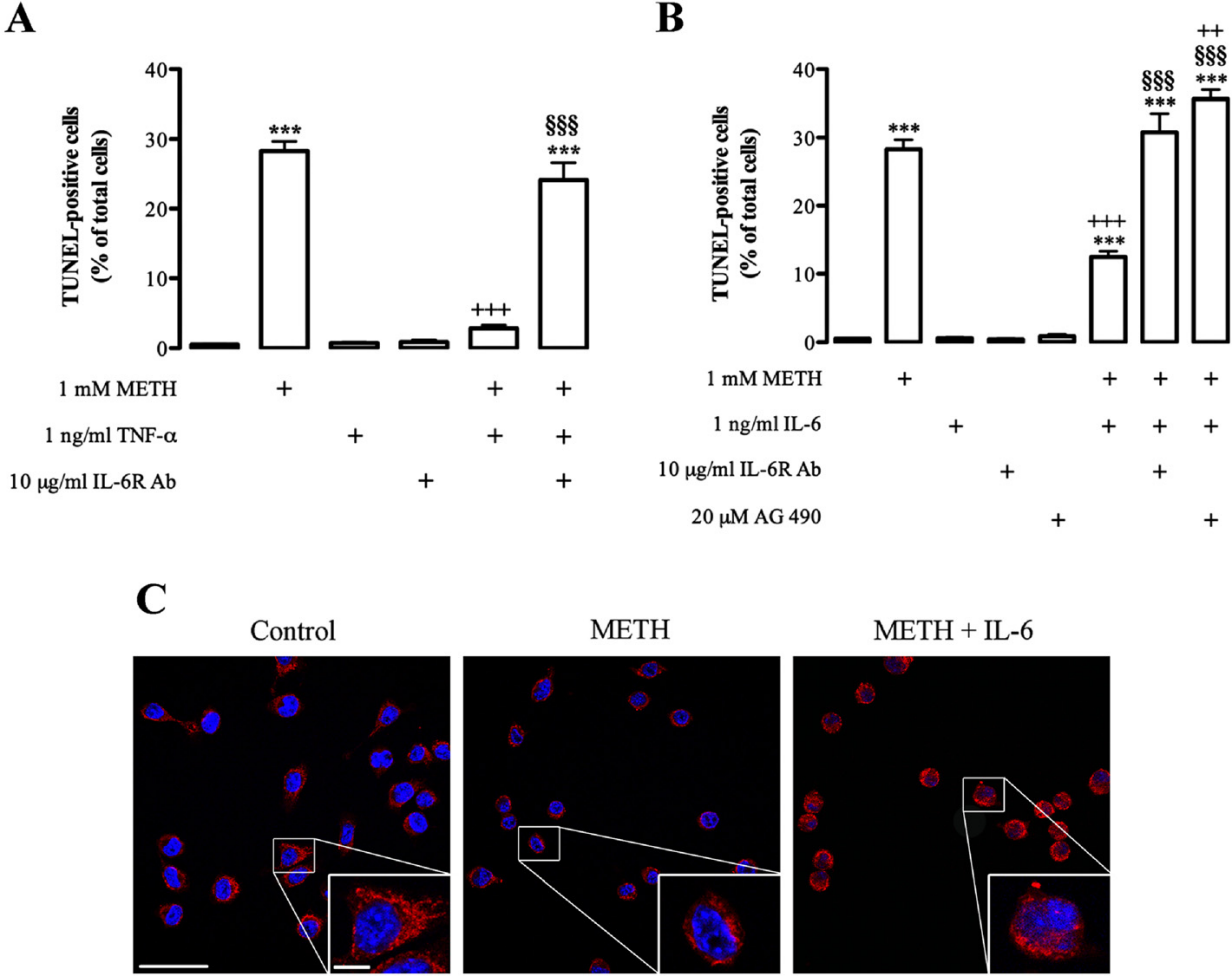
**Protective effect of exogenous TNF-α or IL-6 against METH-induced microglial cell death. (A)** TNF-α (1 ng/mL) completely prevented the increase of TUNEL-positive cells induced by METH; this was abolished by IL-6R antibody (10 μg/mL). **(B)** IL-6 (1 ng/mL) reduced the number of TUNEL-positive cells, and once again the blockade of its receptor with IL-6R antibody abolished this effect. Also, the JAK-STAT3 inhibitor (20 μM AG 490) exacerbated METH toxicity. The results are expressed as mean percentage of total cells ± SEM (n = 17 to 41). ^***^P <0.001, Dunnett’s multiple comparison test, significantly different when compared to control; ^++^*P* <0.01, ^+++^*P* <0.001, Bonferroni’s multiple comparison test, significantly different when compared with 1 mM METH; ^§§§^*P* <0.001, Bonferroni’s multiple comparison test, significantly different when compared with 1 mM METH plus TNF-α or IL-6. **(C)** Representative images of pSTAT3 immunoreactivity (red) in N9 microglial cells under control conditions and exposed to 1 mM METH alone or in the presence of 1 ng/mL IL-6. Cells were also stained with Hoechst 33342 (blue). Scale bar, 20 μm and 50 μm. IL-6: interleukin 6; JAK-STAT: janus kinase-signal transducer and activator of transcription; METH: methamphetamine; pSTAT: phospho-signal transducer and activator of transcription; SEM: standard error of the mean; TNF-α: tumor necrosis factor-alpha; TUNEL: terminal deoxynucleotidyl transferase mediated dUTP nick end labeling assay.

The dual role of cytokines generally depends on the environment, concentration and stimuli duration [50]. Moreover, an insult usually triggers the production of several cytokines that may operate as a cascade [17,51]. Thus, we also aimed to clarify the effect of exogenous TNF-α and IL-6 on METH-induced cell death and the possible interplay between these two cytokines. The concentrations used in the present study were chosen based on previous studies [26,43], but nevertheless we started by demonstrating that they are not toxic to N9 microglial cells by themselves (control: 0.5 ± 0.09%, n = 17; 1 ng/mL; TNF-α: 0.7 ± 0.14%, n = 17, Figure 8A; 1 ng/mL IL-6: 0.6 ± 0.17%, n = 26, Figure 8B). We then showed that 1 ng/mL TNF-α completely prevented the cell death induced by 1 mM METH (METH: 28.28 ± 1.41% of total cells, n = 86; METH + TNF-α: 2.86 ± 0.46% of total cells, n = 25; Figure 8A).

Our next goal was to clarify if the observed protective effect of TNF-α occurred via IL-6 signaling. Thus, we performed the same experiment but in the presence of the IL-6R antibody, and under these conditions TNF-α lost its protective effect (24.13 ± 2.50% of total cells, n = 31; Figure 8A). Based on these observations, IL-6 signaling seems to play an important protective role under conditions of METH toxicity. So, we further evaluated the direct effect of IL-6 (1 ng/mL) and concluded that this cytokine decreased the number of TUNEL-positive cells induced by 1 mM METH to 12.47 ± 0.83% of total cells (n = 44; Figure 8B). Moreover, the blockade of IL-6R by the neutralizing antibody (10 μg/mL IL-6R antibody) abolished the protective effect of IL-6 (30.79 ± 2.74% of total cells, n = 24; Figure 8B). Interestingly, the inhibition of the JAK-STAT3 pathway by 20 μM AG 490 exacerbated the toxic effect of METH (35.7 ± 1.37% of total of cells, n = 18; Figure 8B). To confirm the results above described, particularly the protective role of IL-6 against METH through the activation of the JAK-STAT3 pathway, we performed an immunocytochemistry analysis for pSTAT3. In Figure 8C, it is possible to observe that, following METH treatment, the pSTAT3 levels were decreased, but in the presence of IL-6 there was a recovery of STAT3 phosphorylation levels and also its translocation to the nucleus (Figure 8C). These observations allow us to conclude that exogenous TNF-α is protective against METH-induced microglial cell death and this effect was dependent on IL-6 signaling via JAK-STAT3 pathway activation.

### **The activation of the IL-6 signaling pathway induces alteration in pro-and anti-apoptotic proteins**

**Figure 9:**
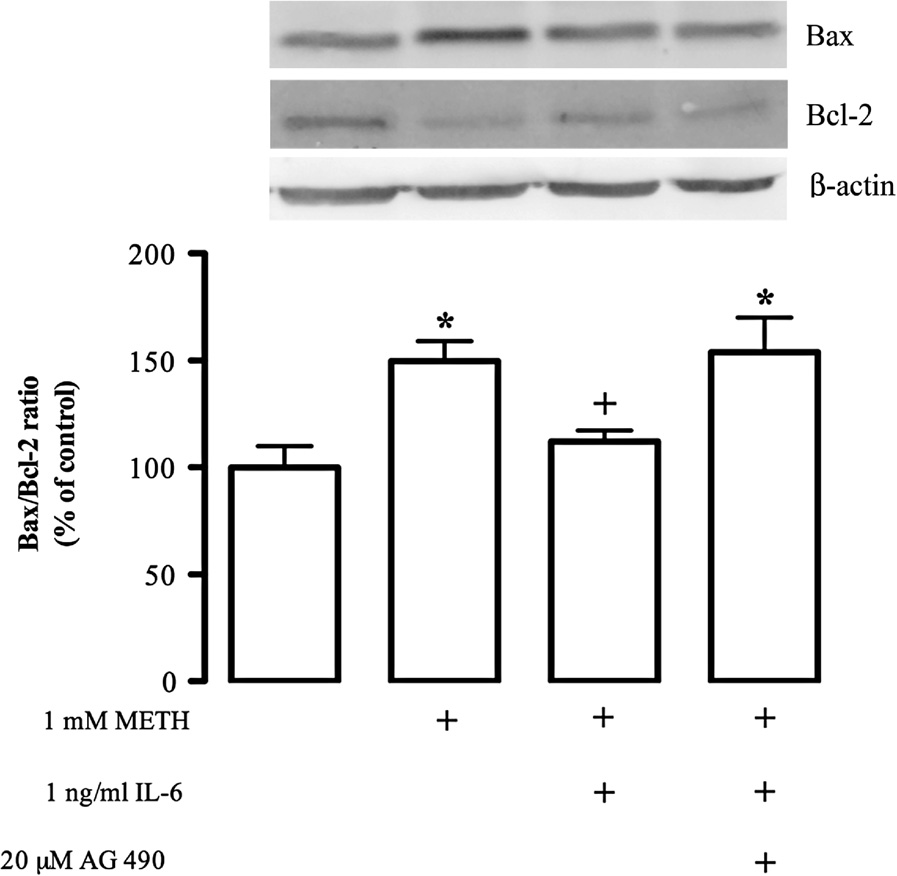
**IL-6 prevents the increase of the Bax/Bcl-2 ratio mediated by METH.** Western blot analysis shows that IL-6 (1 ng/mL) was able to reduce the augmentation of the Bax/Bcl-2 ratio following METH exposure. Above the bars, representative western blot images of Bax (21 kDa), Bcl-2 (26 kDa) and β-actin (42 kDa) are shown. The results are expressed as mean percentage of control ± SEM (n = 4 to 7). ^*^P <0.05, Dunnett’s multiple comparison test, significantly different when compared with control; ^+^*P* <0.05, Bonferroni’s multiple comparison test, significantly different when compared with 1 mM METH. IL-6: interleukin 6; kDa: kiloDaltons; METH: methamphetamine; SEM: standard error of the mean.

Several studies have reported that METH causes significant changes in several pro-and anti-apoptotic proteins, such as Bax, Bad, Bcl-2 and Bcl-XL [52,53]. Furthermore, it has been shown that the JAK-STAT3 pathway is involved in protective mechanisms, in part due to its role in the transcription of anti-apoptotic genes [54]. Hence, we aimed to evaluate if the IL-6 anti-apoptotic effect observed in the present study could be mediated by the up-regulation of anti-apoptotic proteins through the activation of the JAK-STAT3 pathway. For that, the Bax/Bcl-2 ratio was analyzed in microglial cells treated with 1 mM METH alone or in the presence of 1 ng/mL IL-6. We verified that METH increased the Bax/Bcl-2 ratio to 149.8 ± 9.34% of control (n = 7; Figure 9), which is in agreement with our previous results (Figure 1). Furthermore, this ratio was reduced by IL-6 treatment (112.1 ± 5.12% of control, n = 6) to values similar to the control, which supports the hypothesis that the IL-6 signaling pathway mediates anti-apoptotic events. Additionally, to prove the involvement of the JAK-STAT3 pathway on IL-6-induced cell protection, we demonstrated that its inhibition by AG 490 (20 μM) led to a recovery of the Bax/Bcl-2 ratio to levels similar to those observed in the presence of METH (METH + IL-6 + AG 490: 153.9 ± 15.97% of control, n = 6; Figure 9). These data allow us to conclude that the effect of IL-6 was mediated by JAK-STAT3 pathway activation, which culminated in changes of pro-and anti-apoptotic protein levels.

## **Discussion**

It has been extensively described that METH triggers neuronal dysfunction or/and death [1,9,55] and also the activation of glial cells, namely microglia [3,9,10]. However, the role played by proinflammatory cytokines under conditions of METH-induced microglial toxicity is poorly understood. In the present study, we report that METH induces microglial cell death and affects cell proliferation. Furthermore, we also show morphologic alterations in microglia, which are accompanied by an increased in the release and intracellular levels of TNF-α and IL-6. Moreover, for the first time, we demonstrate that the up-regulation of these proinflammatory cytokines does not contribute to METH-induced cell death, and that exogenous TNF-α or IL-6 have a protective effect via activation of the JAK-STAT3 pathway, which in turn leads to a decrease in the Bax/Bcl-2 ratio.

The involvement of inflammatory events, such as gliosis [9,10,13,14] and an increase in the production of proinflammatory cytokines [9,11,13,37], has recently been suggested to play an important role in METH-induced brain dysfunction. Accordingly, our group showed that a single high dose of METH (30 mg/kg; i.p.) led to a rapid up-regulation of both IL-6 and TNF-α mRNA in the mouse hippocampus, frontal cortex and striatum [11]. Moreover, we also demonstrated that the same METH treatment triggered a neuroinflammatory response characterized by microgliosis and astrogliosis, as well as by changes in TNF system protein levels [9]. Concerning *in vitro* studies, there is only one study that has approached this issue by demonstrating that, in highly aggressively proliferating immortalized microglial cells, a non-toxic concentration of METH (0.8 mM, 6 h exposure) increased IL-1β, TNF-α and IL-6 mRNA levels, followed by production of reactive oxygen and nitrogen species [37]. Our data, besides showing that METH can induce microglial cell death by apoptosis, also verified that METH is able to significantly increase the release and production of TNF-α and IL-6. Moreover, the protein levels of TNFR1 and IL-6R-α were also up-regulated, demonstrating that this drug significantly interferes with both proinflammatory cytokine systems. Interestingly, we also found that these alterations occurred at different time-points, since METH-evoked TNF-α release occurred at 1 h, and IL-6 release occurred at 24 h. This time-course raises the hypothesis that the released of TNF-α may stimulate the release of IL-6. In fact, it was demonstrated that TNF-α induces IL-6 production by regulation of its transcription, mainly via TNFR1 [19] through activation of mitogen activated protein kinase/extracellular signal-regulated kinases (MAPK/ERK) and p38 pathways [18,19,24], or also by NFκB cascade [23]. Furthermore, TNF-α is known as a priming cytokine at the apex of inflammatory cascades in immune cells [56]. In agreement, a recent study reported that TNF-null microglia showed a drastic reduction of IL-6 production in response to LPS stimulation [56]. Additionally, Yamashita *et al.*[57], using a co-culture of adipocytes and macrophages, demonstrated that LPS (1 ng/mL) up-regulated IL-6 production, which was partially inhibited by anti-TNF-α neutralizing antibody. Other *in vitro* studies have shown that treatment with TNF-α might be an essential step to IL-6-induced neuroprotection [58], and that TNF-α stimulation increases IL-6R and gp130mRNA expression [58,59]. In agreement with these observations, we showed that TNF-α release evoked by METH preceded the increase of both IL-6 and IL-6R protein levels.

After demonstrating that METH induces significant alterations on microglial TNF-α and IL-6 systems, we further aimed to clarify the role played by such cytokines under conditions of METH-induced cell death. We verified that the blockade of endogenous TNF-α and IL-6 did not affect microglial cell death induced by METH, which suggest that the up-regulation of cytokine release is a consequence of METH toxicity and not a cause. Interestingly, the application of a low concentration of exogenous TNF-α completely prevented apoptotic cell death induced by the drug. In fact, Nakajima *et al.*[60] reported an up-regulation of rat striatal TNF-α levels following a repeated treatment with METH (2 mg/kg for 5 days, subcutaneously), which was associated with a neuroprotective effect. Specifically, they showed that exogenous TNF-α (4 μg; intracerebroventricular administration) blocked locomotor-stimulating and rewarding effects of METH (4 mg/kg; four times at 2 h intervals), and also decreased the extracellular levels of striatal dopamine and potentiated its uptake into synaptosomes [60]. Moreover, we demonstrated that the protective effect of TNF-α occurred via IL-6 signaling pathway, because using an antibody to neutralize the IL-6R or blocking the pathway completely abolished the protective effect.

To date, there is very little information regarding the role of IL-6 under METH toxicity. Ladenheim *et al.*[36] showed that, in IL-6 knockout mice, the neurotoxicity induced by METH was attenuated. The authors demonstrated that IL-6^(−/−)^ mice subjected to a repeated METH treatment (5 or 10 mg/kg; four times at 2 h intervals, i.p.) showed less depletion of dopamine levels and its transporter binding, a reduction in serotonin levels, and also inhibition of gliosis, when compared with wild-type mice [36]. Despite the fact that the protective effect of IL-6 against METH toxicity has never been addressed before, some studies have clearly shown that IL-6 has a protective role under excitotoxic conditions [26,61-63]. In fact, it was demonstrated that the neuroprotective effect mediated by IL-6 against N-methyl-d-aspartate-induced apoptosis of cerebellar granule neurons involves the suppression of intracellular Ca^2+^ overload [61], and was mediated by JAK-STAT3 and PI3K-AKT signaling pathways [61-63]. In line with these observations, we further showed that a low concentration of exogenous IL-6 provided a decrease in the number of apoptotic cells induced by METH, and this effect was mediated via IL-6R activation, since the receptor neutralizing antibody completely blocked its effect. Unexpectedly, the use of AG490 not only abolished the protective effect mediated by IL-6 but also increased the number of apoptotic cells when compared with METH by itself. This observation can be explained by the fact that AG 490 blocked both IL-6R-dependent and -independent activation of JAK-STAT3 pathway. In fact, JAK-STAT3 signaling is important not only to stimulate cellular proliferation and differentiation, but it also plays a central role in cell survival and regeneration in response to several factors, including cytokines [54]. Previous studies showed that STAT3 regulates the transcription of anti-apoptotic genes, such as *bcl-2* and *bcl-xl*[54]. Moreover, STAT3 can also activate the expression of other proteins that belong to the inhibitor of apoptosis protein family, including surviving [64] and cellular inhibitor of apoptosis 2 [65]. Accordingly, here we demonstrated that METH increases the Bax/Bcl-2 ratio and IL-6 is able to completely prevent this effect. Furthermore, when we blocked the JAK-STAT3 pathway, the Bax/Bcl-2 ratio returned to values similar to those observed in the presence of METH. These observations lead us to conclude that the protective effect induced by IL-6 against METH-induced microglial cell death occurs through JAK-STAT3 pathway activation, which in turn interferes with anti- and pro-apoptotic proteins levels. Our results are also in agreement, with the study performed by Cadet *et al.*[66], who showed that overexpression of Bcl-2 protects immortalized rat neural cells against METH-induced apoptosis.

## **Conclusions**

Our results highlight the toxic effect of METH on microglial cells since it induced cell death, affected microglial proliferation and increased the release and intracellular levels of TNF-α and IL-6. Interestingly, endogenous cytokines *per se* did not affect METH-induced cell death, which suggest that alterations in such systems are a consequence rather than a cause of METH-induced microglial apoptosis. Moreover, exogenous low concentrations of TNF-α and IL-6 provided a protective effect via activation of the JAK-STAT3 pathway, which in turn led to changes in the levels of anti-and pro-apoptotic proteins. Thus, our data suggest that IL-6 system is an important target to prevent or at least to minimize the toxic effects of METH.

## **Abbreviations**

ANOVA: analysis of variance; BrdU: 5-bromo-2’-deoxyuridine; BSA: bovine serum albumin; ELISA: enzyme-linked immunosorbent assay; FBS: fetal bovine serum; Iba-1: ionized calcium binding adaptor molecule-1; IL-6: interleukin-6; IL-6R-α: interleukin-6 receptor-alpha; i.p.: intraperitoneal; JAK-STAT3: janus kinase-signal transducer and activators of transcription; kDA: kiloDaltons; LPS: lipopolysaccharide; METH: methamphetamine; NF-kB: nuclear factor kappa-light-chain-enhancer of activated B cells; PBS: phosphate-buffered saline; PFA: paraformaldehyde; RT: room temperature; SEM: standard error of the mean; TNF-α: tumor necrosis factor-alpha; TNFR1: tumor necrosis factor receptor 1; TUNEL: terminal deoxynucleotidyl transferase dUTP nick end labeling; Z-VAD: z-Val-Ala-Dl-Asp-(OMe)-fluoromethylketone.

## Misc

Vanessa Coelho-Santos and Joana Gonçalves contributed equally to this work.

## **Competing interests**

The authors declare that they have no competing interests.

## **Authors’ contributions**

VCS and JG carried out all the experiments. VCS wrote the manuscript and JG designed the figures. APS designed, supervised and secured the funding of the present study. CFR revised the manuscript. All authors have read and approved the final manuscript.
